# Anti-Candidal Activity and Functional Mapping of Recombinant and Synthetic *Neosartorya fischeri* Antifungal Protein 2 (NFAP2)

**DOI:** 10.3389/fmicb.2018.00393

**Published:** 2018-03-07

**Authors:** Liliána Tóth, Györgyi Váradi, Attila Borics, Gyula Batta, Zoltán Kele, Ákos Vendrinszky, Roberta Tóth, Hargita Ficze, Gábor K. Tóth, Csaba Vágvölgyi, Florentine Marx, László Galgóczy

**Affiliations:** ^1^Department of Microbiology, Faculty of Science and Informatics, University of Szeged, Szeged, Hungary; ^2^Doctoral School in Biology, Faculty of Science and Informatics, University of Szeged, Szeged, Hungary; ^3^Department of Medical Chemistry, Faculty of Medicine, University of Szeged, Szeged, Hungary; ^4^Institute of Biochemistry, Biological Research Centre, Hungarian Academy of Sciences, Szeged, Hungary; ^5^Department of Organic Chemistry, Faculty of Science and Technology, University of Debrecen, Debrecen, Hungary; ^6^MTA-SZTE Biomimetic Systems Research Group, University of Szeged, Szeged, Hungary; ^7^Division of Molecular Biology, Biocenter, Medical University of Innsbruck, Innsbruck, Austria

**Keywords:** *Neosartorya fischeri* antifungal protein 2, recombinant protein, protein synthesis, anti-candidal activity, protein structure, functional mapping

## Abstract

The increasing number of life-threatening *Candida* infections caused by antifungal drug-resistant strains urges the development of new therapeutic strategies. The small, cysteine-rich, and cationic *Neosartorya fischeri* antifungal protein 2 (NFAP2) effectively inhibits the growth of *Candida* spp. Limiting factors of its future application, are the low-yield production by the native producer, unavailable information about potential clinical application, and the unsolved relationship between the structure and function. In the present study we adopted a *Penicillium chrysogenum*-based expression system for bulk production of recombinant NFAP2. Furthermore, solid-phase peptide synthesis and native chemical ligation were applied to produce synthetic NFAP2. The average yield of recombinant and synthetic NFAP2 was 40- and 16-times higher than in the native producer, respectively. Both proteins were correctly processed, folded, and proved to be heat-stable. They showed the same minimal inhibitory concentrations as the native NFAP2 against clinically relevant *Candida* spp. Minimal inhibitory concentrations were higher in RPMI 1640 mimicking the human inner fluid than in a low ionic strength medium. The recombinant NFAP2 interacted synergistically with fluconazole, the first-line *Candida* therapeutic agent and significantly decreased its effective *in vitro* concentrations in RPMI 1640. Functional mapping with synthetic peptide fragments of NFAP2 revealed that not the evolutionary conserved antimicrobial γ-core motif, but the mid-N-terminal part of the protein influences the antifungal activity that does not depend on the primary structure of this region. Preliminary nucleic magnetic resonance measurements signed that the produced recombinant NFAP2 is suitable for further structural investigations.

## Introduction

In the last two decades, fungal infections caused by different *Candida* spp. (such as candidemia and candidiasis) have become one of the most frequent healthcare associated infections showing an increasing trend (Lockhart, 2014). In immunocompetent individuals, the members of this genus often cause common but easily-to-treat mucocutaneous candidiasis, but this or an exogenous *Candida* infection can become systemic, associated with high morbidity and mortality rates in immunocompromised patients (Deorukhkar, 2016). However, overall resistance to antifungal agents has remained low within the genus (Lockhart, 2014), a trend in resistance due to misuse of antifungal drugs, especially to azoles and echinocandins has been recently reported; and multidrug resistant strains are also occurring (Bailly et al., 2016; Gonçalves et al., 2016). The antifungal consumption influences the susceptibility profile of *Candida* spp. meaning a selective pressure for the resistant strain (Bailly et al., 2016). There is therefore a need to reduce the application of routinely administered antifungal drugs by introducing novel, alternative agents and therapeutic strategies (e.g., drug combinatory therapy).

Naturally occurring peptides, proteins and their synthetic derivatives with antifungal activity have been proposed as potential sources and templates of novel drugs to treat mycotic infections, and they also have significant commercial potential on the global market for antifungals in the next years (Duncan and O'Neil, 2013). The *Neosartorya fischeri* antifungal protein 2 (NFAP2) is a novel member of small, cysteine-rich, and cationic antifungal proteins from filamentous ascomycetes (Table 1). Within this protein group, NFAP2 and its putative homologs form a phylogenetically distinct clade from the well-characterized *Penicillium chrysogenum* antifungal protein (PAF) and *Penicillium brevicompactum* bubble protein (BP) homologs. Compared to PAF and BP proteins which are mainly effective against molds, NFAP2 shows a unique high anti-yeast activity. Namely, the disulphide-bond stabilized and heat-resistant NFAP2 effectively inhibits the growth of *Candida albicans* and non-*albicans Candida* species *in vitro* possibly due to its prompt membrane disruption ability (Tóth et al., 2016). These last features render NFAP2 exceptionally suitable as potential commercial preservative, bio-pesticide, and drug against yeasts. The yield of NFAP2 in the native producer *N. fischeri* (*Aspergillus fischerianus*) NRRL 181 is quite low (368 ± 19 μg l^−1^; Tóth et al., 2016) from economic view and for further comprehensive and detailed investigations. Therefore, the bulk production of NFAP2 by a “generally recognized as safe” (GRAS) microorganism or development of a chemical strategy for its low-cost synthesis is indispensable.

**Table 1:** Amino acid sequence and *in silico* predicted physical and chemical properties of mature NFAP2 and its peptide fragments.

**Protein/peptide**	**Number of amino acids**	**Molecular weight (kDa)**	**Number of Cys**	**Number of Lys/Arg**	**Theoretical pI**	**Estimated charge at pH 7**	**GRAVY**
**IATSPYYACNCPNNCKHKKGSGCKYHSGPSDKSKVISGKCEWQGGQLNCIAT**							
NFAP2	52	5.6	6	7/0/	9.01	+5.2	−0.731
**KYHSGPSDKSKVISGKC(-SH)EWQGGQLNC(-SH)IAT**							
Fragment 1 (Fr-1)	29	3.1	2	4/0	8.79	+2.1	−0.752
**IATSPYYAC(-SH)NC(-SH)PNNC(-SH)KHKKGSGC(-SH)/SHUFFLE VARIANT: IAGAHKC(-SH)KPC(-SH)YGYKTNSC(-SH)C(-SH)NSPN**							
Fragment 2 (Fr-2, Sh-Fr-2)	23	2.5	4	3/0	8.87	+3.0	−0.704
**GKC(-SH)EWQGGQLNC(-SH)IAT**							
Fragment 3 (Fr-3)	15	1.6	2	1/0	5.99	−0.2	−0.373
**NNC(-SH)KHKKGSGC(-SH)/SHUFFLE VARIANT: C(-SH)C(-SH)NKGKNKGSH**							
Fragment 4 (Fr-4, Sh-Fr-4)	11	1.2	2	3/0	9.39	+3.1	−1.682
**KYHSGPSDKSKVIS**							
Fragment 5 (Fr-5)	14	1.5	0	3/0	9.53	+2.1	−1.157
**IATSPYYAC(-SH)NC(-SH)P**							
Fragment 6 (Fr-6)	12	1.3	2	0/0	5.51	−0.2	+0.192

*GRAVY, grand average of hydropathy value; NFAP2, Neosartorya fischeri antifungal protein 2*.

To meet these requirements, we applied in the present study a recently described *P. chrysogenum*-based expression system (Sonderegger et al., 2016), and a native chemical ligation method (Váradi et al., 2013) to produce recombinant NFAP2 (rNFAP2) and synthetic NFAP2 (sNFAP2). Our further aims were to compare their structure and antifungal activity with those of the native NFAP2 (nNFAP2) from *N. fischeri* NRRL 181. The applied protein synthesis method allowed the functional mapping of NFAP2 to reveal its putative antifungal active site(s). Finally, we investigated the potential clinical applicability of NFAP2 by CLSI-M27A3 susceptibility test method (Clinical and Laboratory Standards Institute, 2008), and its *in vitro* combination with fluconazole (FLC).

## Materials and methods

### Strains and media

*P. chrysogenum* minimal medium (PCMM: 2% sucrose, 0.3% NaNO_3_, 0.05% KCl, 0.05% MgSO_4_ × 7 H_2_O, 0.005% FeSO_4_ × 7 H_2_O (w/v), 0.25% 1 M potassium phosphate buffer pH 5.8, 0.01% trace element solution (v/v); trace element solution: 0.1% FeSO_4_ × 7 H_2_O, 0.9% ZnSO_4_ × 7 H_2_O, 0.4% CuSO_4_ × 5 H_2_O, 0.01% MnSO_4_ × H_2_O, 0.01% H_3_BO_3_, 0.01% Na_2_MoO_4_ × 2 H_2_O (w/v)) was used to produce rNFAP2 by *P. chrysogenum*. To produce ^13^C/^15^N-labeled rNFAP2 the nitrogen and carbon source in PCMM were replaced by 0.3% (w/v) Na^15^NO_3_ and 1% (w/v) ^13^C-glucose (Euriso-Top, Saarbrücken, Germany).

The antifungal activity of recombinant and synthetic NFAP2, and synthetic NFAP2 peptide fragments was investigated against nine yeast isolates (*C. albicans* American Type Culture Collection, Manassas, VA, USA, ATCC 10231; *Candida glabrata* Centraalbureau voor Schimmelcultures, Utrecht, The Netherlands, CBS 138; *Candida guilliermondii* CBS 566; *Candida krusei* CBS 573; *Candida lusitaniae* CBS 6936; *Candida parapsilosis* CBS 604; *Candida tropicalis* CBS 94; *Saccharomyces cerevisiae* Szeged Microbiology Collection, Szeged, Hungary, SZMC 0644; and *Schizosaccharomyces pombe* SZMC 0142). Susceptibility tests were performed in low ionic strength broth medium (LCM: 0.5% glucose, 0.25% yeast extract, 0.0125% peptone (w/v)) and in RPMI 1640 medium (Sigma-Aldrich, St Louis, MO, USA) supplemented with 0.03% (w/v) L-glutamine and buffered to pH 7.0 with 0.165 M 4-morpholinopropanesulfonic acid (Sigma-Aldrich, St Louis, MO, USA).

Yeasts were maintained on yeast extract glucose medium (YEGK: 1% glucose; 1% KH_2_PO_4_; 0.5% yeast extract, 2% agar (w/v)), and *P. chrysogenum* on PCMM supplemented with 2% (w/v) agar at 4°C.

### Recombinant NFAP2 production in *Penicillium chrysogenum*-based heterologous expression system

NFAP2 was produced in *P. chrysogenum* Δ*paf* under the regulation of the strong *paf*-promoter as was described previously (Sonderegger et al., 2016). To generate the NFAP2-producing *P. chrysogenum* strain (*P. chrysogenum nfap2*), first an expression vector was constructed as presented in Supplementary Figure 1. Briefly, the part of the cDNA that codes for the mature *N. fischeri* NRRL 181 antifungal protein (NFAP) fused to *paf* prepro sequence in the pSK275*nfap* vector (Sonderegger et al., 2016) was replaced by the cDNA sequence coding for the mature NFAP2. The *nfap2* cDNA partially overlapping with *paf* pro sequence and *paf* 3′-UTR was amplified (Q5 High Fidelity DNA Polymerase, New England Biolabs, Ipswich, MA, USA) from a synthetic construct inserted in *Eco*RV restriction site of pUC57 (GenScript USA Inc., Piscataway, NJ, USA) using the primers NFAP2F (5′- GGC CGC TGA GGC CGG TGT−3′) and NFAP2R (5′- GGC CAA GCT TCG AGA ATC ACA GG−3′). The 273 bp amplicon contained the *Bbv*CI and *Hind*III restriction sites at 5′ and 3′ends. After enzymatic digestion with *Bbv*CI and *Hind*III (New England Biolabs, Ipswich, MA, USA), the fragment was ligated into the respective restriction sites of a pGEM-T vector (Sonderegger et al., 2016) which already contained the mature NFAP encoding cDNA fused to *paf* prepro sequence flanked with the *paf* 5′- and 3′-UTR regions. With this step, the *nfap* cDNA was replaced to *nfap2* cDNA. The *paf* 5′- and 3′-UTR flanking construct was amplified (Q5 High Fidelity DNA Polymerase, New England Biolabs, Ipswich, MA, USA) from this vector using the primer pair 1stF (5′- GGC CCC ATG GTG GTA AAC AAG TAG TG−3′) / 1stR (5′- GGT GGC GGC CGC TCT AGA ACT AG−3′), and after digestion with *BspM*I, *Not*I (New England Biolabs, Ipswich, MA, USA) the resulted 1033 bp amplicon was inserted into the respectively digested pSK275*paf* vector (Sonderegger et al., 2016) resulting in the expression vector pSK275*nfap2*. Detailed, graphical information is provided in the Supplementary Material (Supplementary Figure 1).

*P. chrysogenum nfap2* was generated by transformation of *P. chrysogenum* Δ*paf* as described (Sonderegger et al., 2016). The gene integration was confirmed by PCR using the above-mentioned primer pairs NFAP2F/NFAP2R, 1stF/1stR, and sequencing of the obtained amplicons.

According to Sonderegger et al. (2016), for recombinant NFAP2 production 2 × 10^8^ conidia of *P chrysogenum nfap2* were cultivated in 200 ml PCMM for 96 h at 25°C with continuous shaking at 200 rpm. NFAP2 was purified from the cell free culture supernatant by cation-exchange chromatography on a Bio-Scale™ Mini Macro-Prep® High S column (Bio-Rad Laboratories, Hercules, CA, USA) using the BioLogic Duo Flow™ system (Bio-Rad Laboratories, Hercules, CA, USA) following the protocol described for native NFAP2 (Tóth et al., 2016). The purity was checked on a 4-12% (w/v) Bis-Tris sodium dodecyl sulfate-polyacrylamide gel (NuPAGE™ Novex™ 4–12% Bis-Tris Protein Gels, 1.5 mm, 10-well; Thermo Fisher Scientific, Waltham, MA, USA) in 1 × MES buffer (Thermo Fisher Scientific, Waltham, MA, USA) using Coomassie Brillant Blue R-250 and silver staining. Protein concentrations were determined spectrophotometrically (A280) considering the respective molar extinction coefficient for NFAP2 (ε = 1.842).

### Synthesis of NFAP2 and its fragments

Fragments of NFAP2 as well as shuffle variants of them were prepared using manual solid-phase peptide synthesis and either Boc or Fmoc chemistry applying N,N'-dicyclohexylcarbodiimide/hydroxybenzotriazole coupling. The thioester part (Fr-2t) was synthesized on a Cys-SH resin as described before (Váradi et al., 2013). Native chemical ligation of Fr-2t and Fr-1 was performed in 0.1 M ammonium acetate buffer (pH 7.5) containing 3% (v/v) thiophenol at 6–8 mg ml^−1^ peptide concentration at 25°C. The reaction was monitored by analytical reversed-phase high performance liquid chromatography (RP-HPLC). Ligation reached completion in 3–4 h in all cases. The precipitate was dissolved by adding 10–15% (v/v) acetonitrile (ACN) and guanidine hydrochloride (6 M final concentration). The reaction mixture was filtered and purified by semi-preparative RP-HPLC. Oxidation of thiols of the purified ligation products was carried out in 0.1 M ammonium acetate buffer (0.2 mg ml^−1^ peptide concentration) having the pH of 7.5 containing 1 mM glutathione (GSH) and 1 mM glutathione disulphide (GSSG). After 24 h reaction time the mixture was injected to semi-preparative RP-HPLC and purified.

### Production of nNFAP2

nNFAP2 as control for experiments was prepared from the supernatant of *N. fischeri* NRRL 181 (Agricultural Research Service Culture Collection, National Center for Agricultural Utilization Research, Peoria, Illinois USA) as described previously (Tóth et al., 2016).

### *In silico* analyses

The molecular weight, pI, grand average of hydropathy (GRAVY) value and total net charge of NFAP2 and its peptide fragments were calculated and predicted by ExPASy ProtParam tool (Gasteiger et al., 2005), and Protein Calculator v3.4 server (The Scripps Research Institute; http://www.scripps.edu/~cdputnam/protcalc.html), respectively. Shuffle variants of Fr-2 and Fr-4 were generated by the random shuffle protein generator of Sequence Manipulation Suite (Stothard, 2000). Secondary structure of NFAP2 was predicted by PSIPRED (v3.3) Protein Analysis Workbench (Buchan et al., 2013).

### Peptide and protein identification

NFAP2 and its peptide fragments were identified by molar mass measurement using a Micromass Q-TOF Premier mass spectrometer (Waters MS Technologies, Manchester, UK) equipped with a nanoelectrospray ion source.

### RP-HPLC purification and analysis

The crude peptides were purified by semipreparative RP-HPLC on a Shimadzu-Knauer apparatus (Kyoto, Japan) using the following solvent system: (A) 0.1% (v/v) trifluoroacetic acid (TFA), (B) 80% (v/v) ACN, 0.1% (v/v) TFA. Linear gradient chosen based on the composition of the peptides was used at the flow rate of 4 ml min^−1^. Peaks were detected at 220 nm. Purity of the peptides was checked by analytical RP-HPLC on an Agilent 1200 Series HPLC instrument using the same solvent system as for purification at 1 ml min^−1^ flow rate.

### Electronic circular dichroism (ECD) spectroscopy

ECD spectroscopic measurements were performed using a Jasco-J815 spectropolarimeter (JASCO Corporation, Tokyo, Japan), in the far-UV range (185–260 nm). Protein samples were dissolved in pure H_2_O in approximately 0.1 mg ml^−1^ concentration and measured in a 0.1 cm pathlength quartz cuvette. First, ECD spectra of samples were recorded at 25°C with a scan speed of 100 nm s^−1^. The temperature was then gradually increased up to 95°C at a rate of 1°C min^−1^ using a Peltier thermoelectronic controller (TE Technology, Traverse City, MI, USA), while ellipticity data was recorded as a function of temperature at 228 nm, appointed by spectra measured at 25°C. The system was allowed to equilibrate for 1 min before measurements were taken at each temperature point.

The obtained melting curves were fitted with an inverse sigmoidal function of which inflection point designated the melting temperature (T_m_) of the native protein structure.

ECD spectra were also recorded at 95°C, the final temperature of the thermal unfolding experiments, then samples were allowed to cool and measured again after 5 min of equilibration at 25°C. The reported spectra are accumulations of 10 scans, from which the similarly recorded, corresponding solvent spectrum was subtracted. Data are given as molar ellipticities. Accurate protein sample concentrations for the corrections of ECD spectra were determined using analytical RP-HPLC.

### Nucleic magnetic resonance (NMR) investigation

NMR spectra were recorded with a Bruker Avance II 500 MHz spectrometer (Billerica, MA, USA), equipped with a 5-mm inverse gradient “txi” probe head. The ^1^H 90 degree pulse was typically 9–12 us, the ^13^C 90 degree pulse 15.7 us, and the ^15^N 90 degree pulse of 37 us. ^13^C-heteronuclear single quantum coherence (HSQC) spectrum of the unlabeled sNFAP2 (4.5 mg dissolved in 275 μl acetate buffer, pH 4.5, 298K) was obtained using the manufacturer's standard “hsqcetgpsi2” pulse program, with time domains of 2,024 and 700 points, and number of scans 64. In case of ^13^C/^15^N-labeled rNFAP2 the pulse program “hsqcctetgpsp” was used for constant time experiment (Vuister and Bax, 1992). ^13^C/^15^N-labeled rNFAP2 (1.1 mg) was dissolved in 275 μl acetate buffer, pH 4.5, 298K for this experiment. Time domains of 2024 and 400 points were used, and the number of scans was 8 at each increment. Full NMR signal assignment and spatial structure determination of NFAP2 is in progress.

### Antifungal susceptibility tests and functional mapping

Antifungal susceptibility tests were performed with nNFAP2, rNFAP2, and sNFAP2 and its peptide fragments in LCM medium against yeast isolates (10^5^ cells ml^−1^) according to the broth microdilution method described by Tóth et al. (2016). Final drug concentration ranged from 25 to 0.098 μg ml^−1^ for rNFAP2 and sNFAP2, and from 100 to 6.25 μg ml^−1^ for the synthetic peptide fragments. The rNFAP2 was tested against nine yeast isolates, while the sNFAP2 and the synthetic peptide fragments against *C. albicans* ATCC 10231, *C. krusei* CBS 573, *C. parapsilosis* CBS 604, and *S. cerevisiae* SZMC 0644.

The CLSI-M27A3 broth microdilution method (Clinical and Laboratory Standards Institute, 2008) was applied to determinate the minimal inhibitory concentrations (MIC) of FLC (Sigma-Aldrich, St Louis, MO, USA) and rNFAP2 against the *Candida* isolates (10^3^ cells ml^−1^) under clinically approved test conditions. For this experiment, the rNFAP2 stock was prepared in bidistilled water, and FLC in dimethyl sulfoxide (DMSO). The investigated concentration range was 64–0.0625 μg ml^−1^ for FCL and 100–0.39 μg ml^−1^ for rNFAP2.

Antifungal synergism between rNFAP2 and FLC were investigated using the checkerboard microdilution method (Eliopoulos and Moellering, 1996) in RPMI 1640 medium applying the drug stock preparation method and cultivation conditions described in CLSI-M27A3 document (Clinical and Laboratory Standards Institute, 2008). *C. albicans, C. parapsilosis*, and *C. krusei* (10^3^ cells ml^−1^) were involved in the combination tests. rNFAP2 in final concentrations ranging from 100 to 6.25 μg ml^−1^ was mixed with the FLC in final concentrations ranging from the respective MIC of FLC to its fifth two-fold dilution step. The fractional inhibitory concentration index (FICI) as a measure of synergism and antagonism was calculated (Johnson et al., 2004). Synergism was defined as FICI≤0.5, indifference as 0.5 < FICI ≤ 4 and antagonism was defined when FICI > 4 (Odds, 2003).

LCM plates were incubated at 30°C, while RPMI 1640 plates at 35°C for 48 h without shaking, then the absorbance (OD_620_) was measured with a microtiter plate reader (SPECTROstar Nano, BMG Labtech, Ortenberg, Germany) after resuspension of the cultures with pipette. The absorbance of the untreated control cultures was referred to 100% growth in each case. MIC was defined as the lowest antifungal protein, peptide or drug concentration at which growth was not detected (growth ≤ 5%) compared to the untreated control.

All susceptibility tests were repeated at least two times in three replicates.

### Propidium iodide (PI) staining

Plasma membrane disrupting activity of NFAP2 and its peptide fragments was investigated by applying the membrane impermeant, red-fluorescent nuclear and chromosome stain PI. Mid-log phase *C. albicans* ATCC 10231 cells (2 × 10^7^ cells ml^−1^) were treated with MICs of nNFAP, Fr-2-4, and Sh-Fr-2 and 4 (Table 2) in LCM for 10 min at 30°C with continuous shaking at 200 rpm. After the treatment, cells were washed with LCM, and then stained with 5 μg ml^−1^ PI for 10 min at room temperature in the dark, and then they were washed again with LCM and resuspended in final volume of 100 μl LCM. Cells treated with 70% (v/v) ethanol for 10 min at 4°C were used as positive staining control. Untreated cells were used as negative staining control.

**Table 2:** Minimal inhibitory concentrations of nNFAP2, rNFAP2, sNFAP2 and its peptide fragments in LCM after incubation for 48 h at 30°C.

	**MIC (μg ml^−1^)**										
**Protein, peptide / Fungus**	**nNFAP2**	**rNFAP2**	**sNFAP2**	**Fr-1**	**Fr-2**	**Sh-Fr-2**	**Fr-3**	**Fr-4**	**Sh-Fr-4**	**Fr-5**	**Fr-6**
*Candida albicans* ATCC 10231	6.25	6.25	6.25	>100	50	50	>100	50	50	>100	>100
*Candida glabrata* CBS 138	1.56	1.56	n.d.	n.d.	n.d.	n.d.	n.d.	n.d.	n.d.	n.d.	n.d.
*Candida guilliermondii* CBS 566	1.56	1.56	n.d.	n.d.	n.d.	n.d.	n.d.	n.d.	n.d.	n.d.	n.d.
*Candida krusei* CBS 573	12.5	12.5	12.5	100	50	50	>100	50	50	>100	>100
*Candida lusitaniae* CBS 6936	3.125	3.125	n.d.	n.d.	n.d.	n.d.	n.d.	n.d.	n.d.	n.d.	n.d.
*Candida parapsilosis* CBS 604	1.56	1.56	1.56	>100	50	50	>100	50	50	>100	>100
*Candida tropicalis* CBS 94	1.56	1.56	n.d.	n.d.	n.d.	n.d.	n.d.	n.d.	n.d.	n.d.	n.d.
*Saccharomyces cerevisiae* SZMC 0644	3.125	3.125	3.125	100	50	50	>100	50	50	>100	>100
*Schizosaccharomyces pombe* SZMC 0142	1.56	1.56	n.d.	n.d.	n.d.	n.d.	n.d.	n.d.	n.d.	n.d.	n.d.

*Fr-1 — Fr-6, synthetic peptide fragments 1-6 of Neosartorya fischeri antifungal protein 2 (Table 1 and Figure 3), MIC, minimal inhibitory concentration, n.d., not determined, nNFAP2, native NFAP2 produced by N. fischeri NRRL 181, rNFAP2, recombinant NFAP2 produced by P. chrysogenum nfap2, Sh-Fr-2 and Sh-Fr-4, shuffle variant of Fr-2 and Fr-4s, NFAP2, synthetic NFAP2*.

### Microscopy

Yeast cells were visualized by light and fluorescence microscopy (Carl Zeiss Axiolab LR 66238C; Zeiss, Oberkochen, Germany) and photographed with a microscope camera (Zeiss AxioCam ERc 5s; Zeiss, Oberkochen, Germany).

### Statistical analysis

Microsoft Excel 2010 software (Microsoft, Edmond, WA, USA) was used to calculate standard deviations.

## Results

### Generation of rNFAP2 and sNFAP2

**Figure 1:**
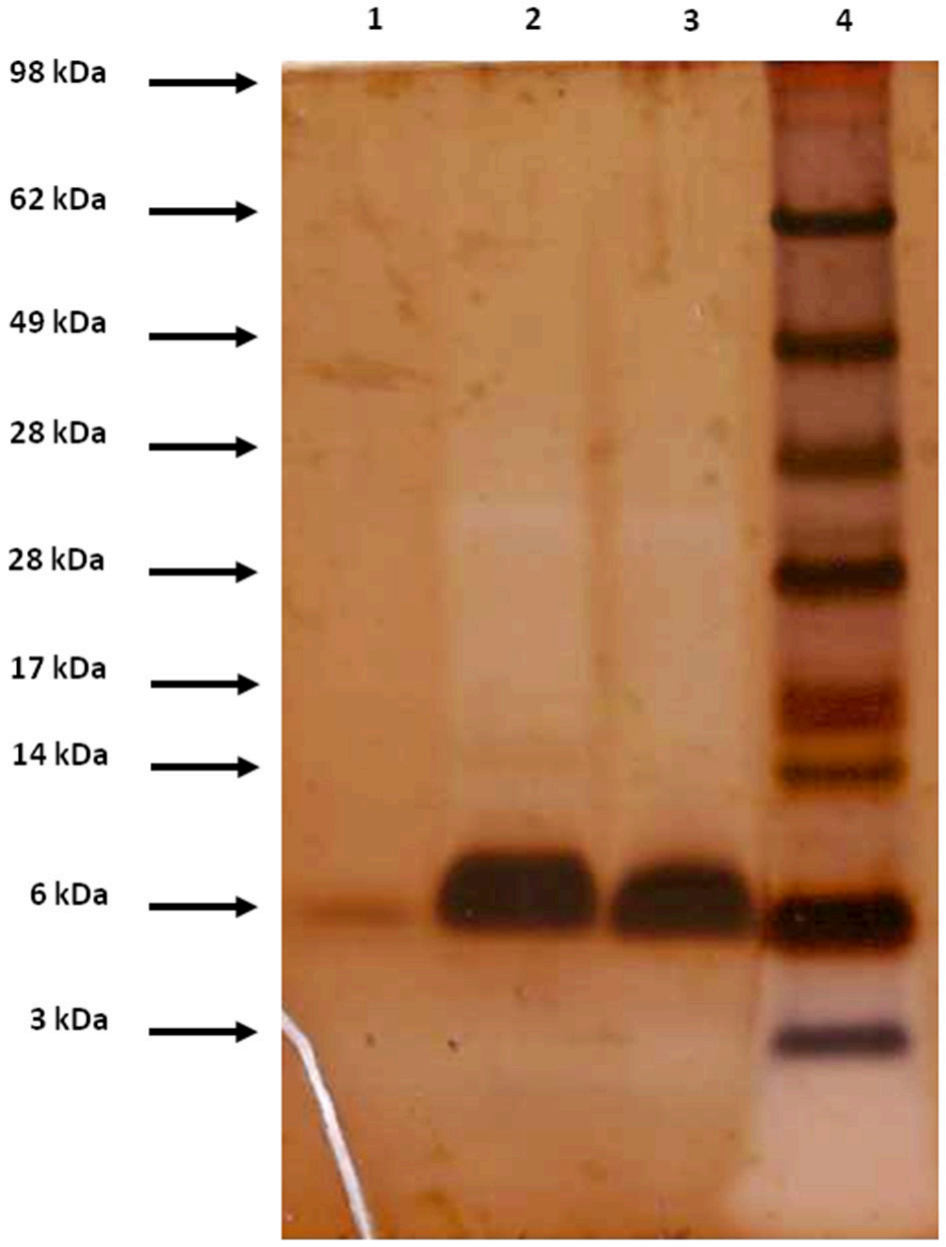
Appearance of nNFAP2, rNFAP2, and sNFAP2 in 4-12% (w/v) Bis-Tris sodium dodecyl sulfate-polyacrylamide gel (NuPAGE™ Novex™ 4–12% Bis-Tris Protein Gels, 1.5 mm, 10-well; Thermo Fisher Scientific, Waltham, MA, USA) in 1×NuPAGE™ MES running buffer (Thermo Fisher Scientific, Waltham, MA, USA). Protein bands are visualized applying silver staining. Lane 1: nNFAP2 (2 μg), lane 2: rNFAP2 (10 μg), lane 3: sNFAP2 (10 μg), lane 4: SeeBlue™ Plus2 Pre-stained Protein Standard (Thermo Fisher Scientific, Waltham, MA, USA). nNFAP2: native NFAP2 produced by *N. fischeri* NRRL 181, rNFAP2: recombinant NFAP2 produced by *P. chrysogenum nfap2*, sNFAP2: synthetic NFAP2.

**Figure 2:**
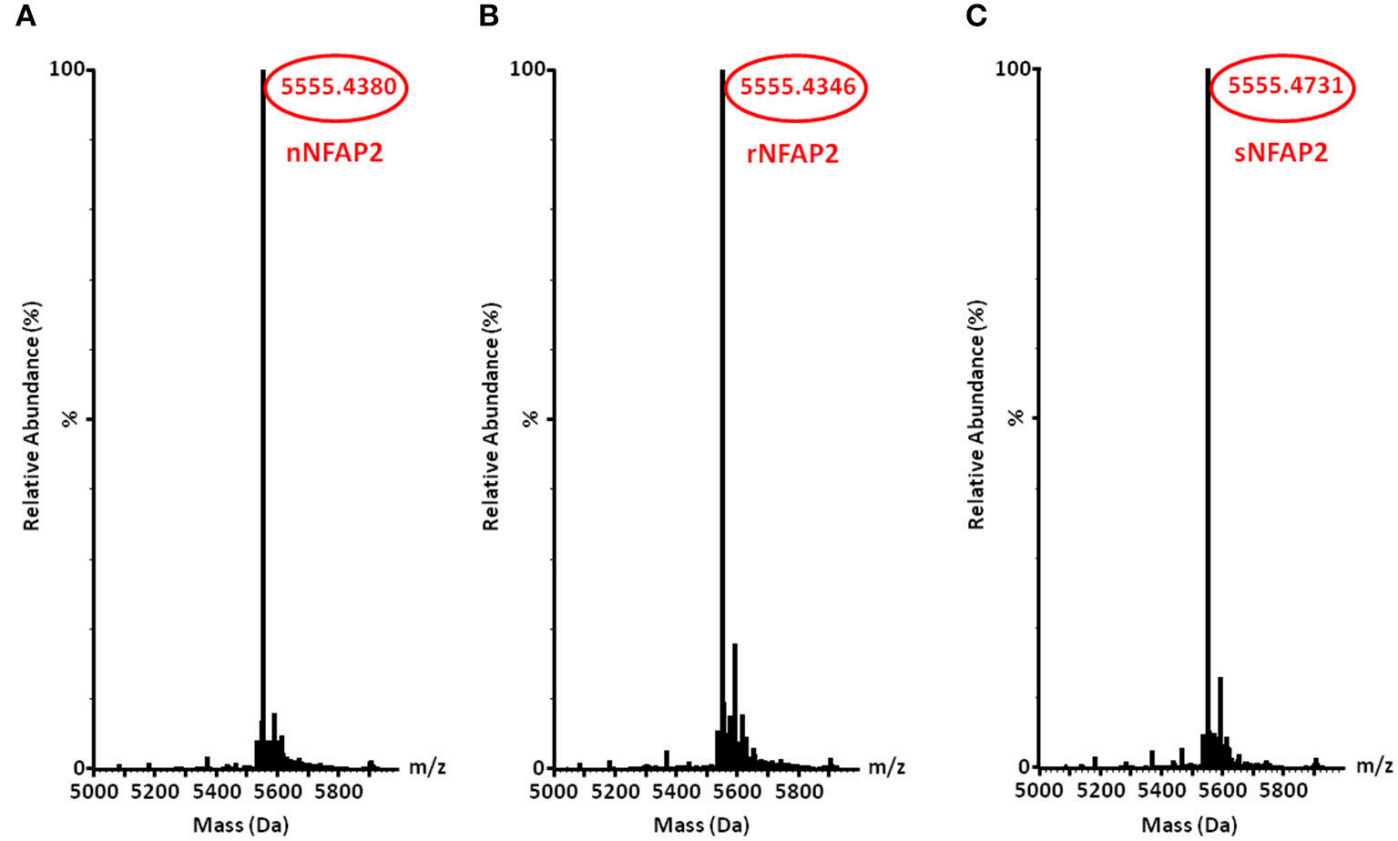
Monoisotopic molecular mass (+H^+^) of **(A)** nNFAP2, **(B)** rNFAP2 and **(C)** sNFAP2. nNFAP2: native NFAP2 produced by *N. fischeri* NRRL 181, rNFAP2: recombinant NFAP2 produced by *P. chrysogenum nfap2*, sNFAP2: synthetic NFAP2.

The rNFAP2 was secreted into the supernatant of the *P. chrysogenum nfap2* strain, from where it was purified to homogeneity. Only one protein band was detected by sodium-dodecyl-sulfate polyacrylamide gel electrophoresis, which corresponded to the molecular weight of nNFAP2 (Figure 1). Electrospray ionization mass spectrometry (ESI-MS) analysis confirmed that the monoisotopic molecular mass of this protein (5555.4346 Da) corresponded well to the detected monoisotopic molecular mass of nNFAP2 (5555.4380 Da) (Tóth et al., 2016) (Figures 2A,B). This result unambiguously proved the heterologous production of correctly processed NFAP2 with three intramolecular disulphide bonds between the six cysteine residues. The final rNFAP2 yield was 15 ± 1.2 mg l^−1^ (*n* = 2).

**Figure 3:**
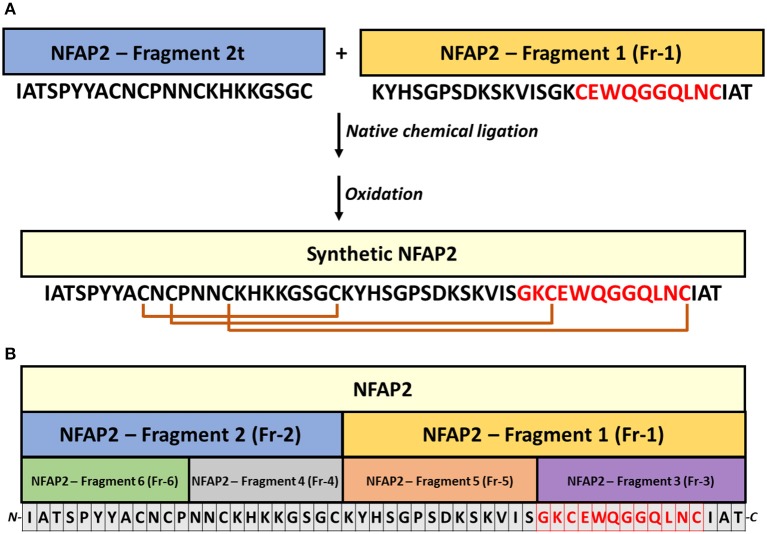
Synthesis strategy of NFAP2 and its peptide fragments. **(A)** Two synthetic peptides, Fragment 2t (thioester form), and Fragment 1, were linked by native chemical ligation (Váradi et al., 2013) to produce full-length synthetic NFAP2. **(B)** The synthetized peptide fragments for functional mapping cover the full-length protein. Intramolecular disulphide bridges between cysteines are marked with orange lines and the γ-core motif in red letters.

For the chemical synthesis of NFAP2, the C-terminal half of NFAP2 (Fragment 1, Fr-1) was prepared, and in addition to this, the N-terminal half of the protein was synthesized in thioester form (Fragment 2t, Fr-2t) to allow native chemical ligation with Fr-1 (Váradi et al., 2013) (Figure 3A). Cysteine thiols of the ligation products were oxidized to disulphide bridges with glutathione redox buffer having the GSH:GSSG ratio of 1:1. ESI-MS analysis revealed the expected molecular mass for sNFAP2, having three intramolecular disulphide bonds (5555.4731 Da in Figure 2C). sNFAP2 showed the same migration profile as nNFAP2 and rNFAP2 in protein gel (Figure 1). The yield of reduced NFAP2 in native chemical ligation was 40–42%, and that of the oxidized protein found to be 23–25%. The overall yield of sNFAP2 was reduced by four HPLC purification steps to 2–3%. The average of 5–6 mg of pure sNFAP2 could be produced from altogether 280 mg of Fr-1 and Fr-2t.

### Structural analysis of rNFAP2 and sNFAP2

#### RP-HPLC analysis

**Figure 4:**
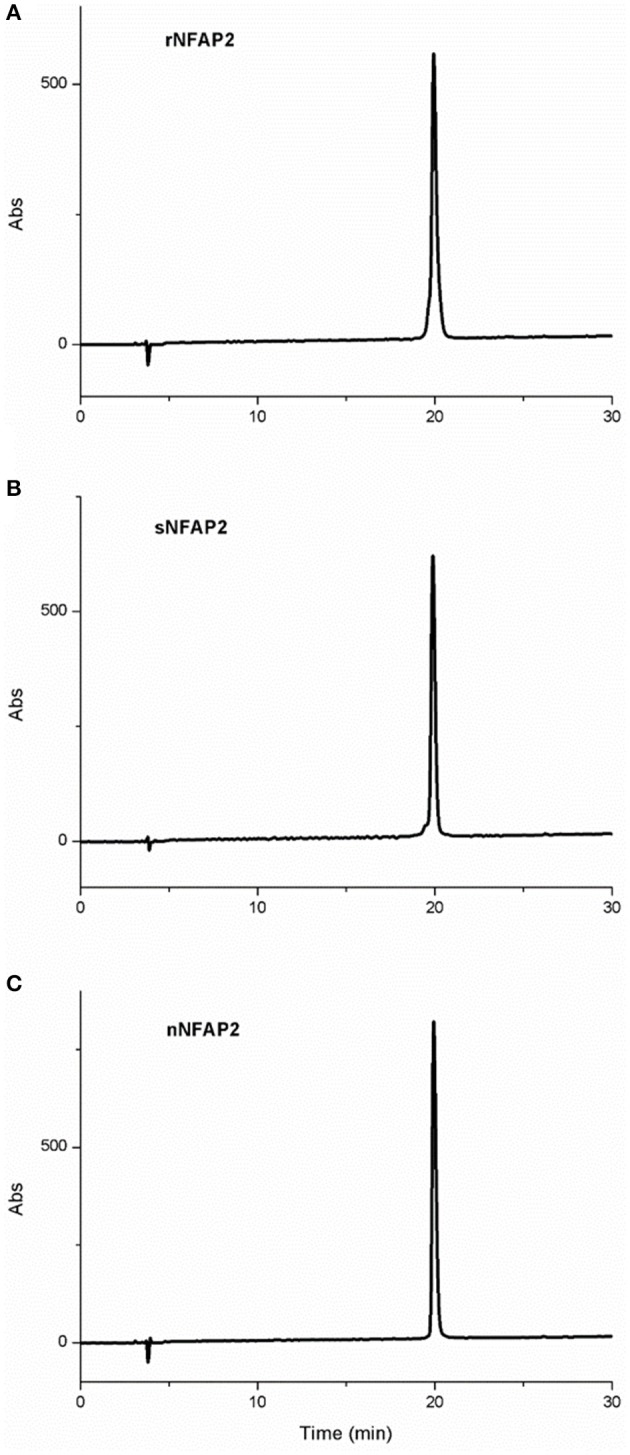
RP-HPLC chromatogram of **(A)** rNFAP2, **(B)** sNFAP2, and **(C)** nNFAP2. A linear gradient was applied from 5 to 35% (v/v) solvent (B) in 30 min. nNFAP2: native NFAP2 produced by *N. fischeri* NRRL 181, rNFAP2: recombinant NFAP2 produced by *P. chrysogenum nfap2*, sNFAP2: synthetic NFAP2.

RP-HPLC is an appropriate tool for the investigation of structural features of proteins related to disulphide bond pattern. Formation of interlocking disulphide bridges characteristic for small, cysteine-rich antifungal proteins from filamentous ascomycetes results in decreased retention time on a reversed-phase column. RP-HPLC revealed the same retention time for the rNFAP2 and sNFAP2 as for the native one confirming right pairing of cysteines and native fold of the recombinant and synthetic proteins (Figure 4). The RP-HPLC purified protein samples were used in the further structural investigation experiments.

#### ECD spectroscopy

**Figure 5:**
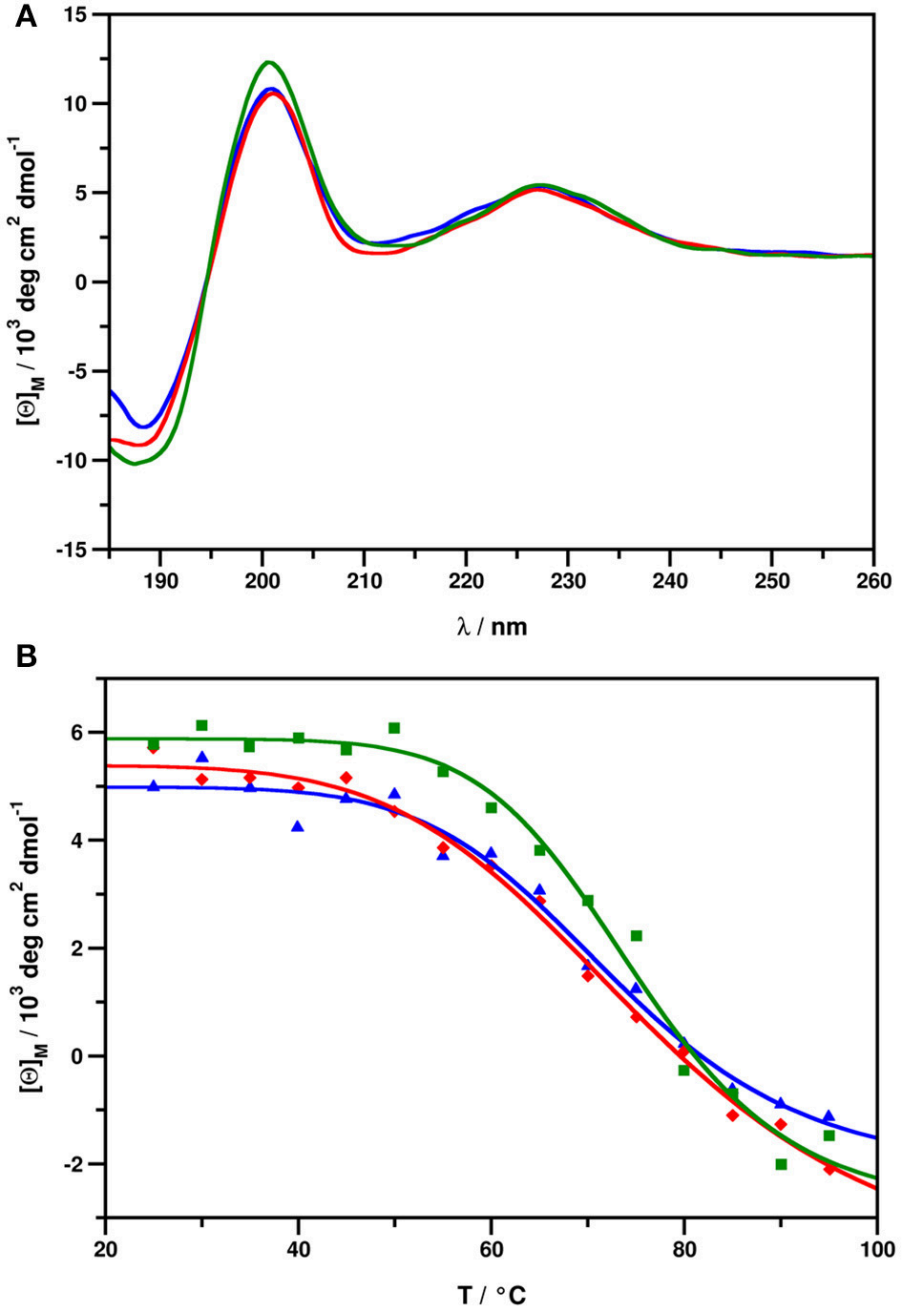
**(A)** ECD spectra of nNFAP2 (blue), rNFAP2 (red), and sNFAP2 (green), recorded at 25°C. **(B)** Thermal unfolding curves measured at 228 nm for nNFAP2 (blue, T_m_ = 72.92°C, *R*^2^ = 0.9837), rNFAP2 (red, T_m_ = 75.79°C, *R*^2^ = 0.9942), and sNFAP2 (green, T_m_ = 74.94°C, *R*^2^ = 0.9864). nNFAP2: native NFAP2 produced by *N. fischeri* NRRL 181, rNFAP2: recombinant NFAP2 produced by *P. chrysogenum nfap2*, sNFAP2: synthetic NFAP2.

ECD spectra of all NFAP2 samples (Figure 5A) show features highly similar to those reported earlier for this class of proteins (Fizil et al., 2015; Sonderegger et al., 2016; Tóth et al., 2016; Galgóczy et al., 2017; Garrigues et al., 2017), with contributions emerging from β-conformation (200 nm, 212 nm) and disulphide bridges (228 nm). In fact, all NFAP2 samples possessed identical secondary structural elements regardless of their native, recombinant or synthetic origin (Figure 5A). Thermal unfolding experiments followed by ECD indicated, that the native folds of nNFAP2, rNFAP2, and sNFAP2 remain intact up to approximately 70°C (Figure 5B), and thermal denaturation is reversible (Supplementary Figure 2). This contradicts previous observations taken for nNFAP2 samples purified by dialysis, where only partial structural reorganization was observed, even 4 weeks after the annealing (Tóth et al., 2016). This discrepancy may be attributed to higher integrity of samples purified by RP-HPLC as opposed to those purified by dialysis.

#### NMR measurements

**Figure 6:**
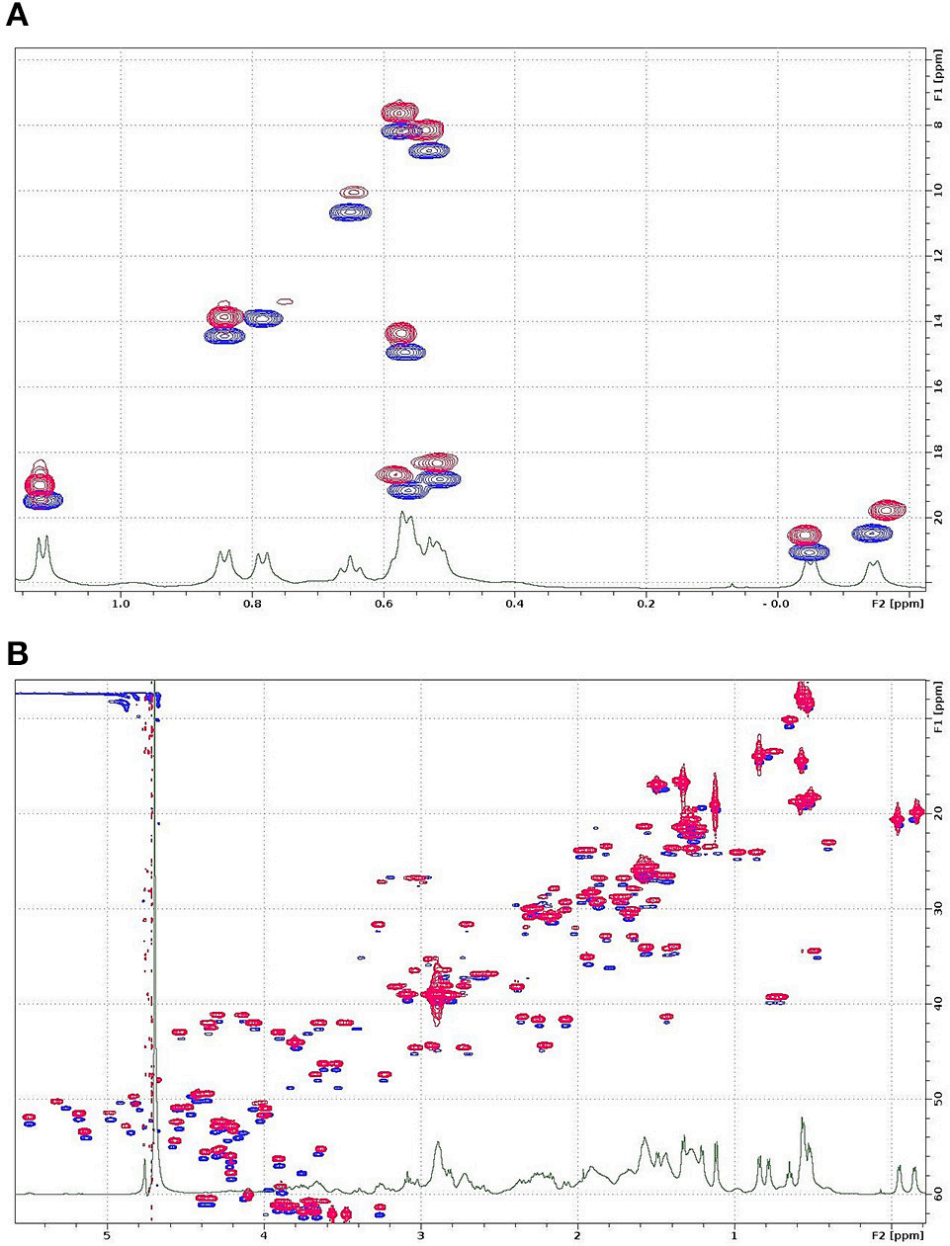
^13^C-^1^H HSQC spectra of rNFAP2 and sNFAP2: **(A)** Methyl and **(B)** aliphatic CH region of rNFAP2 (red) and sNFAP2 (blue). Overlaid spectra are intentionally shifted in the vertical ^13^C axis direction for better visibility. rNFAP2: recombinant NFAP2 produced by *P. chrysogenum nfap2*, sNFAP2: synthetic NFAP2.

NMR investigations require milligrams of isotope-labeled protein to reveal the tertiary structure and structural dynamics. Hence, the production of isotope-labeled NFAP2 by the native producer *N. fischeri* NRRL 181 is not economic because of the low protein yield and expense of isotope labeled compounds. Considering the data collected with ESI-MS, RP-HPLC, and ECD that revealed very similar structures of sNFAP2, rNFAP2 and nNFAP2, we started the preliminary NMR investigations with unlabeled sNFAP2 and ^13^C/^15^N-labeled rNFAP2. First, we confirmed the structure identity of sNFAP2 and rNFAP2 using the ^13^C-HSQC type fingerprint spectra of the two compounds. The methyl region of sNFAP2 (blue in Figure 6A) and rNFAP2 (red, intentionally shifted up in Figure 6A) are expanded and overlaid, while Figure 6B shows a bigger range of the aliphatic CH region. Though the signal intensities are different due to different relaxation behavior and the constant-time version of HSQC experiment necessary to remove ^13^C-^13^C couplings in the rNFAP2, it can be seen that nearly all HSQC peak has a pair, a closest neighbor in the map. This observation strongly suggests, that the two compounds have identical constitution and even more very similar, folded spatial structure. Considering these results, the ^13^C/^15^N-labeled rNFAP2 is a suitable object to reveal the folded structure and structural dynamics of NFAP2. The NMR assignment and 3D structure determination of NFAP2 is in progress.

### Antifungal activity of rNFAP2 and sNFAP2

#### Antifungal activity in LCM

nNFAP2, rNFAP2, and sNFAP2 totally inhibited the yeast growth after 48 h at their applied concentration range, and there was no difference between their MICs (Table 2). The MICs varied in the range of 1.56–12.5 μg ml^−1^ depending on the tested species. Both, the rNFAP2 and the sNFAP2 showed dose-dependent antifungal activity (Supplementary Figures 3A,B). However, sNFAP2 was tested against only four species because of its low-yield production, but based on the results of the susceptibility tests (Table 2) we expect the same MICs to nNFAP2 and rNFAP2 against the other non-investigated isolates. Our results propose the potential clinical applicability of the high-yield produced rNFAP2.

#### Antifungal activity in RPMI 1640

To determine MICs of rNFAP2 under standardized clinical microbiological conditions, its *in vitro* antifungal activity was investigated against human pathogenic *Candida* spp. in human inner fluid mimicking RPMI 1640 medium following the recommendations of CLSI-M27A3 susceptibility test method (Clinical and Laboratory Standards Institute, 2008). MICs are summarized in Table 3. *C. albicans, C. krusei, C. lusitaniae*, and *C. tropicalis* showed higher MICs than 100 μg ml^−1^, however this applied concentration could reduce the fungal growth to 30 and 50% except of *C. krusei* (Supplementary Figure 4). The growth of *C. glabrata, C. guilliermondii*, and *C. parapsilosis* was completely inhibited in the investigated concentration range with MICs of 12.5, 3.125, and 100 μg ml^−1^, respectively (Table 3). These results sign the limited clinical applicability of NFAP2 as monotherapeutic and systemic antifungal drug. Thus, its efficacy in combinatorial drug application was investigated.

**Table 3:** Minimal inhibitory concentrations of rNFAP2 and FLC, and their combination in RPMI 1640 after incubation for 48 h at 35°C.

**Protein, peptide / Species**	**MIC (μg ml^−1^)**	**FICI**	**Effect in combination**			
	**rNFAP2(a)**	**FLC(a)**	**rNFAP2(c)**	**FLC(c)**		
*Candida albicans* ATCC 10231	200^*^	1	6.25	0.25	0.28	synergistic
*Candida glabrata* CBS 138	12.5	n.d.	n.d.	n.d.	n.d.	n.d.
*Candida guilliermondii* CBS 566	3.125	n.d.	n.d.	n.d.	n.d.	n.d.
*Candida krusei* CBS 573	400^*^	64	6.25	64	0.52	indifferent
*Candida lusitaniae* CBS 6936	>100	n.d.	n.d.	n.d.	n.d.	n.d.
*Candida parapsilosis* CBS 604	100	8	6.25	1	0.19	synergistic
*Candida tropicalis* CBS 94	>100	n.d.	n.d.	n.d.	n.d.	n.d.

* *The individual MIC above 100 μg ml^−1^ was determined to calculate FICI for the respective strains. (a): MIC of the drug alone, (c): MIC of the drug in combination. FICI: fractional inhibitory concentration index, FLC: fluconazole, MIC: minimal inhibitory concentration, n.d.: not determined, rNFAP2: recombinant NFAP2 produced by P. chrysogenum nfap2*.

### Combination of rNFAP2 with FLC

*C. albicans* and *C. parapsilosis* as the most prevalent opportunistic pathogen of adults and neonates, and *C. krusei* as the most frequent azole-resistant species (Guinea, 2014) were involved in the combination tests under the same conditions described in the CLSI-M27A3 method (Clinical and Laboratory Standards Institute, 2008). The combination of rNFAP2 with FLC, the first-line therapeutic drug in treatment of *Candida* infections (Lockhart, 2014) was studied. To calculate the fractional inhibitory concentration index (FICI) to reveal the synergism between the compounds, exact MICs of rNFAP2 and FLC were determined (Table 3). Co-administration of rNFAP2 and FLC acted synergistically (FICI = 0.28 and 0.19) against *C. albicans* and *C. parapsilosis* (Table 3), while it was indifferent (FICI = 0.52) at *C. krusei* (Table 3). The effective combinations of rNFAP2 and FLC are shown in Supplementary Figure 5.

### Functional mapping of NFAP2

Peptide motifs derived from a full-length antifungal protein allow the identification of putative antimicrobial active motifs (Garrigues et al., 2017). Considering this approach, we involved six synthetic peptide fragments (Fr 1-6) of NFAP2 (Fragments 1–6 in Table 1 and Figure 3B) in antifungal activity assays to identify the functional active site(s) of the protein. Solid-phase peptide synthesis was applied to prepare peptide fragments of NFAP2. Two halves (Fr-1 and Fr-2 in Table 1) and four quarters (Fr-3-6 in Table 1) were synthesized on a solid support applying either Boc or Fmoc chemistry (Figure 3B). Besides this, shuffle variants of Fr-2 and Fr-4 (Sh-Fr-2 and Sh-Fr-4 in Table 1) were prepared using the same method. Peptides were purified by preparative RP-HPLC. Analytical RP-HPLC showed high purity of the products, and ESI-MS confirmed the expected molecular mass of them (data not shown).

**Figure 7:**
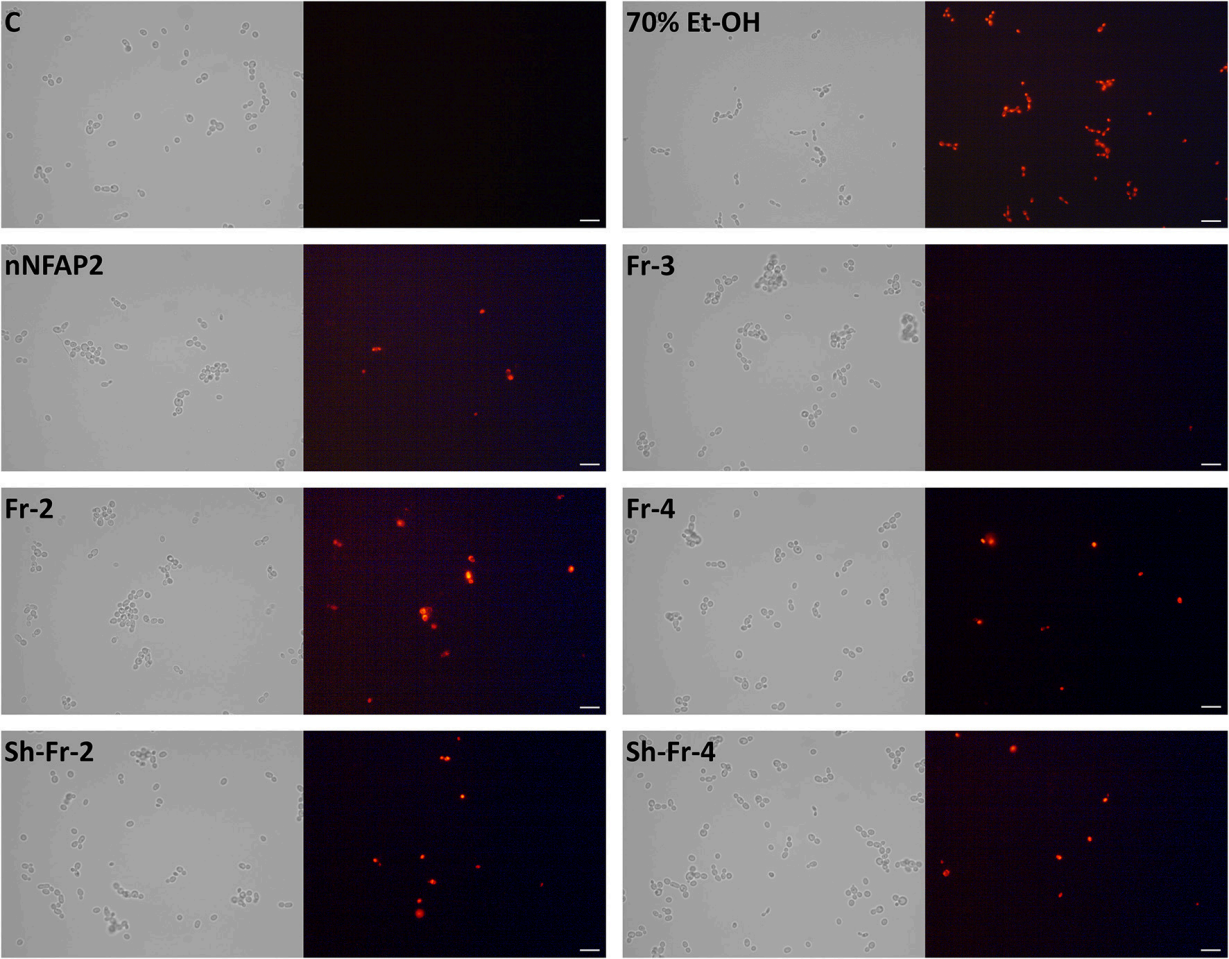
PI staining of *C. albicans* ATCC 10231 cells after treatment at MIC (Table 2) of nNFAP2 and its synthetic peptide fragments (Fr-2 - 4 and Sh-Fr-2 and 4) for 10 min at 30°C. Scale bars represent 20 μm. 70% (v/v) Et-OH: positive PI-staining control, C: untreated, negative control, Fr-2 - 4: synthetic peptide Fragment 2 - 4 of NFAP2 (Table 1), nNFAP2: native NFAP2 produced by *N. fischeri* NRRL 181, Sh-Fr-2 and 4: shuffle variants of synthetic peptide Fragment 2 and 4 of NFAP2 (Table 1).

In LCM, only Fr-2 and Fr-4 showed dose-dependent antifungal activity (Supplementary Figures 6A,C), and their MICs were much higher than the full-length NFAP2 (Table 2). At their MICs they exerted the prompt plasma membrane disruption effect on *C. albicans* (Figure 7) what we already observed at nNFAP2 by applying PI staining (Tóth et al., 2016). Taking into account that the Fr-4 is the C-terminal part of the Fr-2, and the N-terminal Fr-6 part of Fr-2 did not show antifungal activity (Figure 3B and Table 2), we assume that the mid-N-terminal part of the protein (Fr-4 in Figure 3B) influences the antifungal activity.

Previous studies demonstrated that synthetic peptide fragments of antifungal proteins and their rational-designed variants show remarkable inhibitory potential on fungi if they are hydrophilic and have high positive net-charge (Sagaram et al., 2011; Garrigues et al., 2017). Therefore, we were curious whether the observed antifungal activity of Fr-2 and Fr-4 depends on these features or the primary structure. Hence, we also involved the shuffle variants of these two peptides (Sh-Fr-2 and Sh-Fr-4 in Table 1) in antifungal activity assays. Both shuffle variants showed the same inhibitory potential as Fr-2 and Fr-4 (Supplementary Figures 6B,D, Table 2) and the prompt plasma membrane disruption effect (Figure 7). We conclude that the antifungal activity of NFAP2 does not depend on the primary structure of the mid-N-terminal region, but possibly on the net charge and hydrophilicity.

The C-terminal part of NFAP2 contains the consensus γ-core motif [GXC]-[X_3−9_]-[C] (Figure 3A), which is important for the activity or folding of antifungal proteins from animals, humans, and antifungal plant defensins (Lacerda et al., 2014). Interestingly, the peptide fragment containing the γ-core motif of NFAP2 (*N-*GKCEWQGGQLNC*-C*) (Fr-3 in Figure 3B) was inactive against yeasts (Table 2) and did not disrupt the plasma membrane (Figure 7) indicating that this specific motif alone has no anti-yeast function, but presumably needs the structural-functional support from other parts of NFAP2. The experimental proof of this hypothesis is in progress.

## Discussion

In the present study, we generated functional recombinant and synthetic NFAP2 which underlines the applicability of heterologous expression and chemical synthesis in the production of cysteine-rich antifungal proteins. Recombinant production of the NFAP by the *P. chrysogenum*-based expression system has been reported recently, however the NFAP yield reached only three-times that of the native producer (Sonderegger et al., 2016). In the present study, the average yield of rNFAP2 was approx. 40-times higher than in *N. fischeri* NRRL 181 (Tóth et al., 2016). It is worth to mention that the applied *P. chrysogenum* Q176 strain is recognized as GRAS organism by the US Food and Drug Administration. However, chemical synthesis of ascomycetous cysteine-rich antifungal proteins has been described previously: functional PAF was produced by solid-phase peptide synthesis and native chemical ligation by Váradi et al. (2013). Here, we show out that this synthesis method is also suitable to generate other functional and phylogenetically distinct cysteine-rich antifungal protein from PAF, the NFAP2.

The correct folding is mandatory for full antifungal activity. This correlation was proved for NFAP (Galgóczy et al., 2017). The data collected with ESI-MS, RP-HPLC, ECD and NMR verified the correct processing and folding of rNFAP2 and sNFAP2. Considering the structural identity between the rNFAP2 and nNFAP2, the ^13^C/^15^N-labeled rNFAP2 is a suitable object to analyse the tertiary structure and folding dynamics of NFAP2, which is currently in progress.

The antifungal activity of recombinant and synthetic NFAP2 was the same to the native protein and they exerted fungicidal activity on yeasts after 2 days of exposure. This observation and the easy production of high-yield rNFAP2 could open new perspectives for its medical application as a novel antifungal agent to treat problematic *Candida* infections caused by strains resistant to clinically administrated antifungal drugs. However, rNFAP2 showed remarkable antifungal effect in LCM, its activity dramatically decreased in the complex RPMI 1640 medium (Tables 2, 3). As RPMI 1640 mimics the composition of human fluids, systemic application of NFAP2 as monotherapy is questionable, but the topical application might be successful as suggested for PAF (Galgóczy et al., 2008). In agreement with our observation, high MICs in RPMI 1640 medium for PAF and NFAP have been reported against human pathogenic dermatophytes (Galgóczy et al., 2008), aspergilli and fusaria (Virágh et al, 2014). The reason for this phenomenon could be the high cation concentration of this medium which reduces the antifungal efficacy of PAF (Kaiserer et al., 2003; Sonderegger et al., 2017) and NFAP (Galgóczy et al., 2017).

Combinatorial application of antifungal agents with different mode of action and metabolism is taken into consideration when the infective fungus shows low susceptibility or resistance to one of them, and/or prolonged sole drug administration in high-dosage can cause severe side-effects in the host. Co-administration strategy allows reduction in drug dosage, can shorten the period of effective treatment and decrease the risk of side-effects. FLC is inexpensive, readily available and the most frequently prescribed antifungal agent worldwide to treat *Candida* infections. However, an emergence in the number of less susceptible or resistant *Candida* strains has been recently reported (Bailly et al., 2016; Gonçalves et al., 2016). The combination of NFAP2 with FLC reduced its effective *in vitro* dosage in a synergistic or additive way, as demonstrated for PAF in a previous study (Galgóczy et al., 2008). Based on these promising results, the FLC-antifungal protein combination could represent a novel therapeutic strategy in the treatment of fungal infections. Detailed *in vivo* pharmacotherapy and clinical studies are required to prove this assumption.

Functional mapping of a protein provides a platform to identify functionally active sites of the molecule which is indispensable for its rational design to develop novel antifungal molecules with improved activity, efficacy and selectivity. In antifungal plant defensins the presence of the consensus [GXC]-[X_3−9_]-[C] γ-core motif determines the antimicrobial activity (Lacerda et al., 2014). A high number of hydrophilic, positively charged amino acids in the γ-core motif of defensins promote cell-killing, while more hydrophobic and negatively charged amino acids trigger morphogenic effect on hyphae. Interestingly, synthetic plant defensin peptides that span the γ-core show the same antimicrobial effect as the full-length proteins (Sagaram et al., 2011). Membrane disruption activity of hydrophilic and cationic antimicrobial peptides on *Candida* spp. has been already proved (Nawrot et al., 2013; Swidergall and Ernst, 2014). Functional mapping of NFAP2 identified the functionally active motif *C-*NNCKHKKGSGC*-N* to reside in the mid-N-terminal part of the protein, not to γ-core motif *C-*GKCEWQGGQLNC*-N* that localizes in the C-terminus. This C-terminal part of the protein is almost neutrally charged (estimated net charge at pH = 7.0 is −0.2) and slightly hydrophilic (GRAVY value is −0.993) in contrast to the mid-N-terminal part which contains several positively charged (estimated net charge at pH = 7.0 is +3.1) and hydrophilic amino acids (GRAVY value is −1.682). *In silico* structural prediction indicated that this part of the protein forms a surface accessible, elongated and flexible loop region that protrudes from a compact tertiary structure (Supplementary Figure 7). This and the PI-staining results (Figure 7) let us purpose that the *C-*NNCKHKKGSGC*-N* region determines the membrane disruption ability of NFAP2 (Tóth et al., 2016). The shuffle variant of this protein region also disrupted the *C. albians* cell membrane within short exposure time (Figure 7) indicating that the high positive charge and hydrophilicity could be the key to the cell-killing effect, not the primary structure. It is worth to mention that ECD spectra of synthetic NFAP2 fragments indicated the absence of stable secondary structural elements both for the antifungal inactive and active ones (Supplementary Figure 8).

In conclusion, our study significantly contributes to a better understanding of the structure-function relationship of NFAP2 and related proteins, which is a prerequisite for their possible application as antifungal agents or the development of new antifungal strategies against *Candida* infections in the future.

## Author contributions

GKT, CV, FM, and LG: conceived and supervised the study, designed experiments, and edited the manuscript; LG: performed *in silico* predictions, cloning, transformation; LT and RT: performed protein preparation, *in vitro* antifungal susceptibility tests, functional mapping, analysis of related data; GV and ÁV: performed protein and peptide synthesis; GV: performed RP-HPLC analysis, and analysis of related data; ZK: performed identification of proteins by mass spectrometry and analysis of related data; AB and HF: performed ECD spectroscopy and analysis of related data; GB: performed NMR spectroscopy and analysis of related data; LT: performed investigation of antifungal mechanism and related microscopy; GV, AB, GB, ZK, FM, and LG: wrote the manuscript and made manuscript revisions. All authors read and approved the submitted version.

### Conflict of interest statement

The authors declare that the research was conducted in the absence of any commercial or financial relationships that could be construed as a potential conflict of interest.
