# Supplementary material for: Anti-Candidal Activity and Functional Mapping of Recombinant and Synthetic Neosartorya fischeri Antifungal Protein 2 (NFAP2)
Source: Front Microbiol. 2018 Mar 7;9:393. doi: 10.3389/fmicb.2018.00393 (PMC5845869; doi:10.3389/fmicb.2018.00393)
Supplement: Supplementary file 1 [file Presentation1.pdf]

Supplementary Material

Anti-candidal activity and functional mapping of recombinant and synthetic *Neosartorya fischeri* antifungal protein 2 (NFAP2)

Liliána Tóth, Györgyi Váradi, Attila Borics, Gyula Batta, Zoltán Kele, Ákos Vendrinszky, Roberta Tóth, Hargita Ficze, Gábor K. Tóth, Csaba Vágvölgyi, Florentine Marx, László Galgóczy*

*** Correspondence:** László Galgóczy: galgoczi@gmail.com

1 Supplementary Figures and Tables

1.1 Supplementary Figures

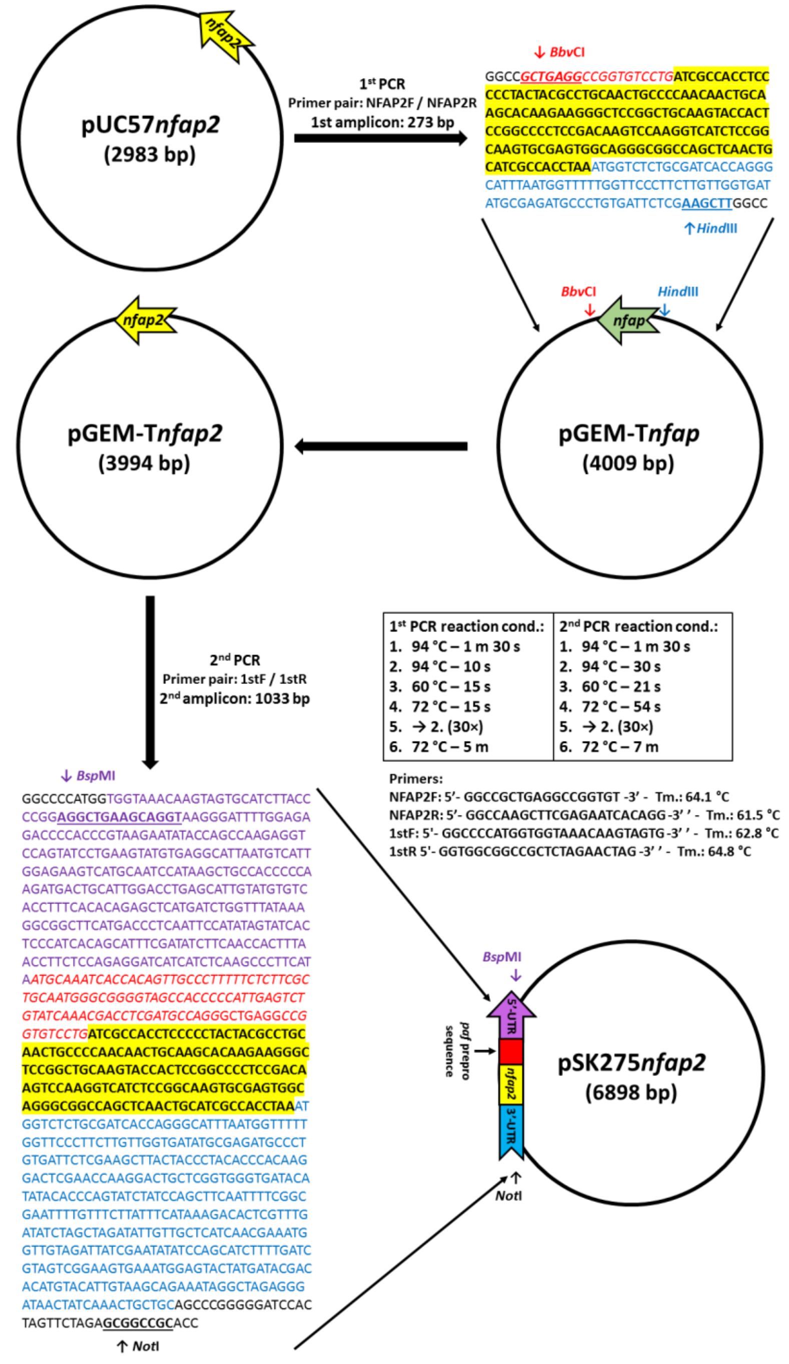

Supplementary Figure 1. Schematic representation of the cloning strategy of the NFAP2 expression vector pSK275*nfap2*. The mature NFAP2 coding cDNA is highlighted with yellow background, the *paf* prepro sequence indicted red letters, 5’- and 3’-UTR are show by lilac and blue letters. The restriction sites are underlined and shown with vertical bold arrows.

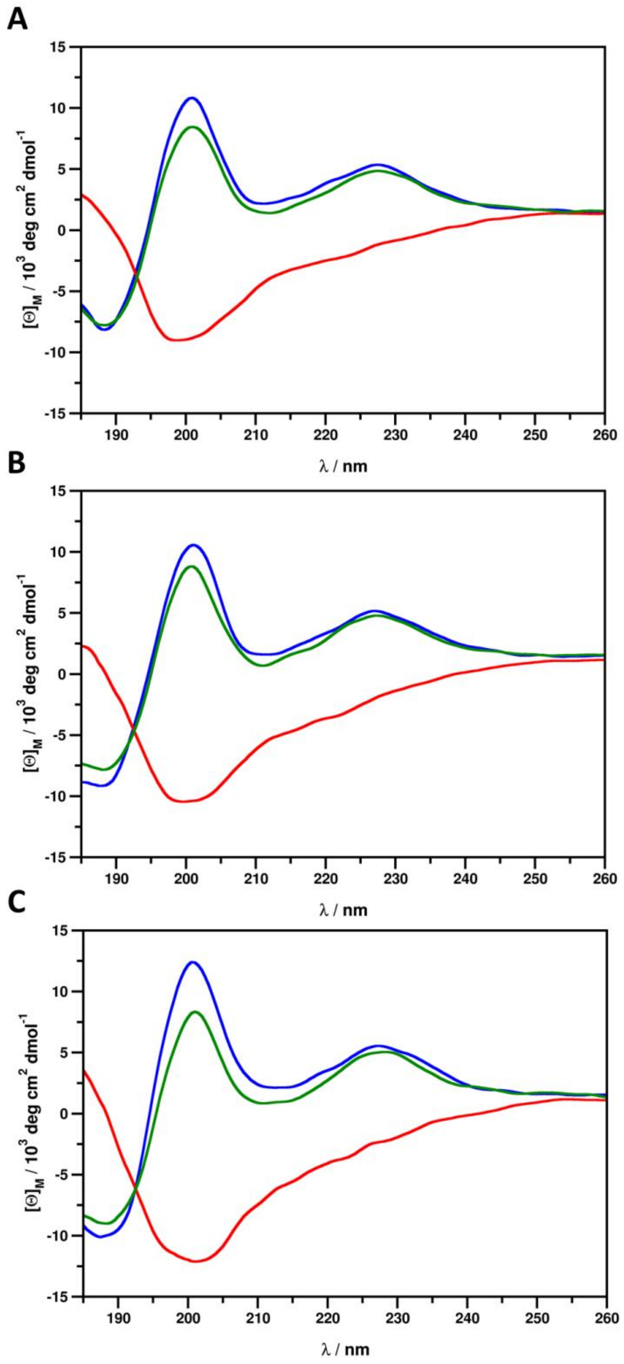

Supplementary Figure 2. ECD spectra of **(A)** nNFAP2, **(B)** rNFAP2 and **(C)** sNFAP2 recorded at 25 °C (blue), 95 °C (red), and after cooling and 5 minutes of equilibration at 25 °C (green). nNFAP2: native NFAP2 produced by *N. fischeri* NRRL 181, rNFAP2: recombinant NFAP2 produced by *P. chrysogenum nfap2,* sNFAP2: synthetic NFAP2.

A

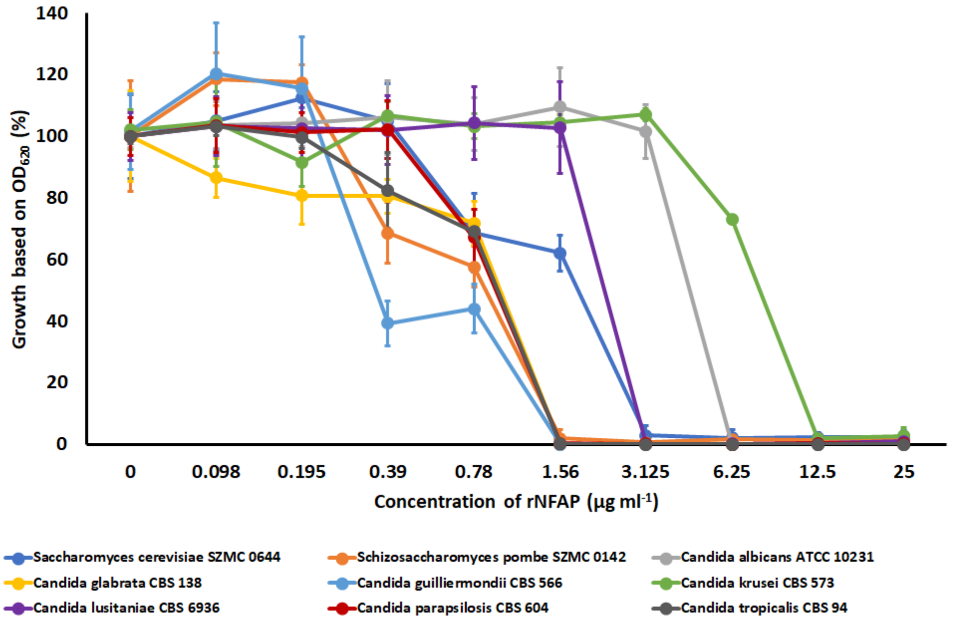

B

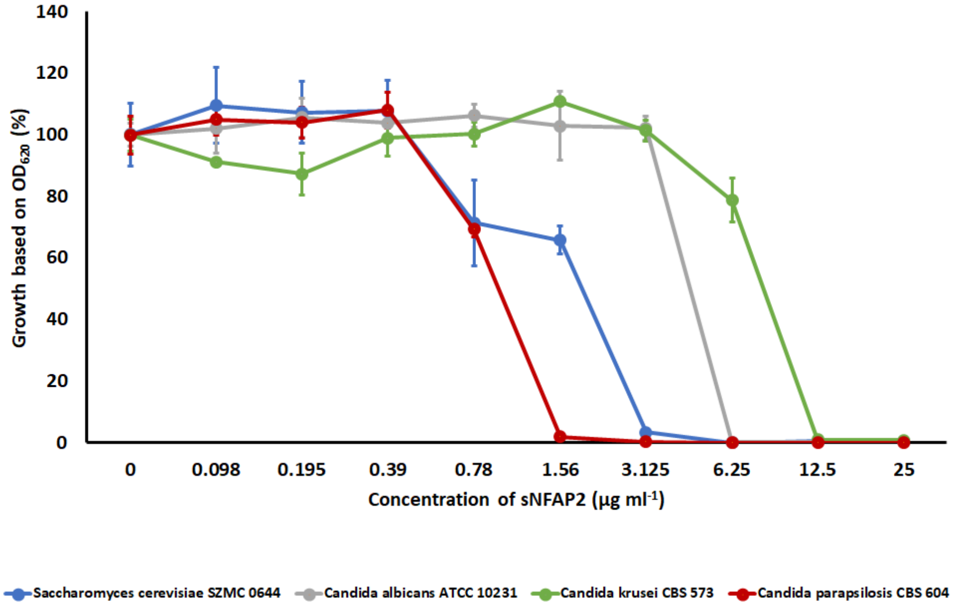

Supplementary Figure 3. Growth inhibition potential of **(A)** rNFAP2 and **(B)** sNFAP2 at different concentrations in LCM after incubation for 48 hours at 30 °C. The untreated control was defined as 100% of growth. rNFAP2: recombinant NFAP2 produced by *P. chrysogenum nfap2*, sNFAP2: synthetic NFAP2.

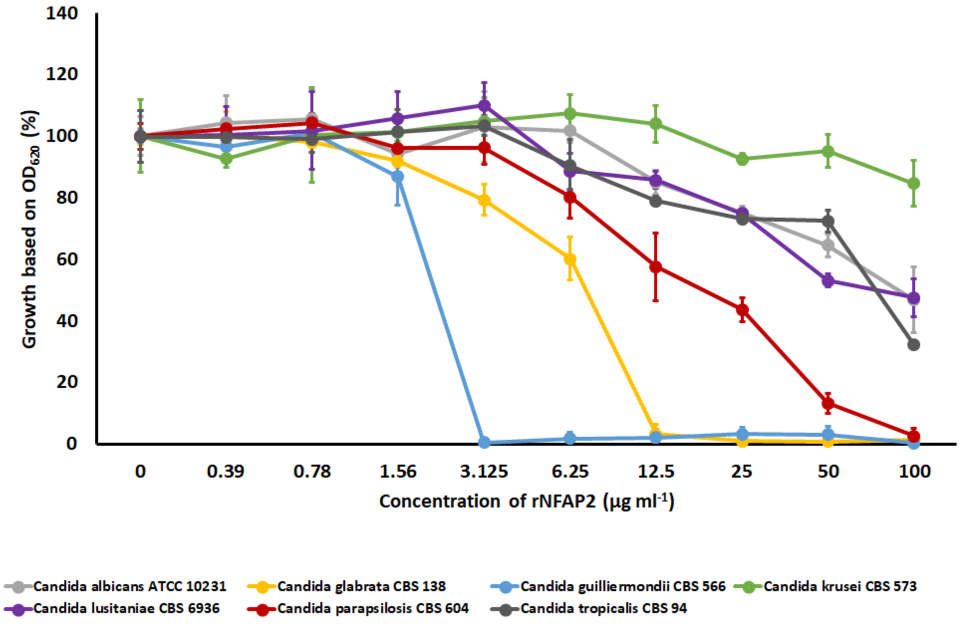

Supplementary Figure 4. Growth inhibition potential of rNFAP2 at different concentrations in RPMI 1640 medium after incubation for 48 hours at 35 °C. rNFAP2: recombinant *Neosartorya fischeri* antifungal protein 2. The untreated control was defined as 100% of growth. rNFAP2: recombinant NFAP2 produced by *P. chrysogenum nfap2*.

A

Candida albicans ATCC 10231						
**rNFAP2 / FLC**	**100**	**50**	**25**	**12.5**	**6.25**	**0**
**1**	0.25±0.36	0.59±0.83	0.67±0.95	0.34±0.47	0.84±1.18	1.17±0.95
**0.5**	0.42±0.59	1.68±0.71	1.01±1.42	1.51±1.18	3.43±2.72	9.11±0.35
**0.25**	1.51±1.18	3.43±107	4.62±1.55	9.46±1.30	10.55±1.42	21.52±8.17
**0.125**	20.85±4.38	31.49±2.13	29.23±3.67	25.29±1.90	50.92±6.40	60.44±3.12
**0.0625**	32.69±0.98	54.05±0.08	58.59±0.88	67.24±0.60	71.47±0.01	81.02±3.48
**0**	43.22±4.50	65.66±2.37	97.04±9.78	98.69±0.90	104.56±1.85	100.28±0.40

B

Candida krusei CBS 573						
**rNFAP2 / FLC**	**100**	**50**	**25**	**12.5**	**6.25**	**0**
**64**	0.65±0.61	1.85±0.15	1.09±0.00	1.09±0.31	1.74±0.31	0.54±0.46
**32**	3.26±3.07	15.54±3.23	19.13±2.46	27.83±0.61	33.8±1.38	66.96±4.30
**16**	51.09±2.46	45.48±2.83	52.5±0.15	59.67±2.31	67.04±1.41	89.24±2.00
**8**	107.61±4.92	107.28±0.15	107.83±2.46	94.35±9.84	100.65±1.38	100.76±2.61
**4**	105.96±4.18	107.07±0.15	101.41±1.38	105.54±3.54	104.78±1.23	97.17±5.23
**0**	100±15.99	99.24±12.14	100.11±2.31	105.33±3.23	109.13±2.15	103.59±5.07

C

Candida parapsilosis CBS 604						
**rNFAP2 / FLC**	**100**	**50**	**25**	**12.5**	**6.25**	**0**
**8**	4.35±1.02	1.67±0.71	3.99±0.51	2.54±0.51	2.90±1.02	3.73±0.05
**4**	3.93±0.58	5.43±0.51	3.26±0.51	4.05±0.42	2.87±1.63	7.9±1.02
**2**	2.54±2.56	5.8±2.05	2.9±1.02	2.54±1.54	3.78±1.24	27.17±7.69
**1**	5.8±2.05	6.16±1.54	2.90±1.03	2.54±2.56	6.23±1.64	73.41±2.77
**0.5**	5.07±1.02	8.48±1.74	14.86±4.61	24.86±1.33	78.26±3.07	107.17±1.33
**0**	2.54±1.54	6.52±2.05	35.29±2.36	103.48±3.48	101.52±0.10	104.78±6.76

Supplementary Figure 5. Growth percentages (%) of **(A)** *C. albicans* ATCC 10231, **(B)** *C. krusei* CBS 573, and **(C)** *C. parapsilosis* CBS 604 in combinatorial application of rNFAP2 and fluconazole in RPMI 1640 medium after incubation for 48 hours at 35 °C. **(A,B,C)** Red cells indicated the total growth inhibition (growth < 5%), orange cells the reduced growth ability (growth between 5% and 90%), green cells the total growth (growth between 90% and 100%). The untreated control was defined as 100% of growth. The effective concentration combinations below the individual MICs are framed with bold line. FLC: fluconazole, rNFAP2: recombinant NFAP2 produced by *P. chrysogenum nfap2*.

A

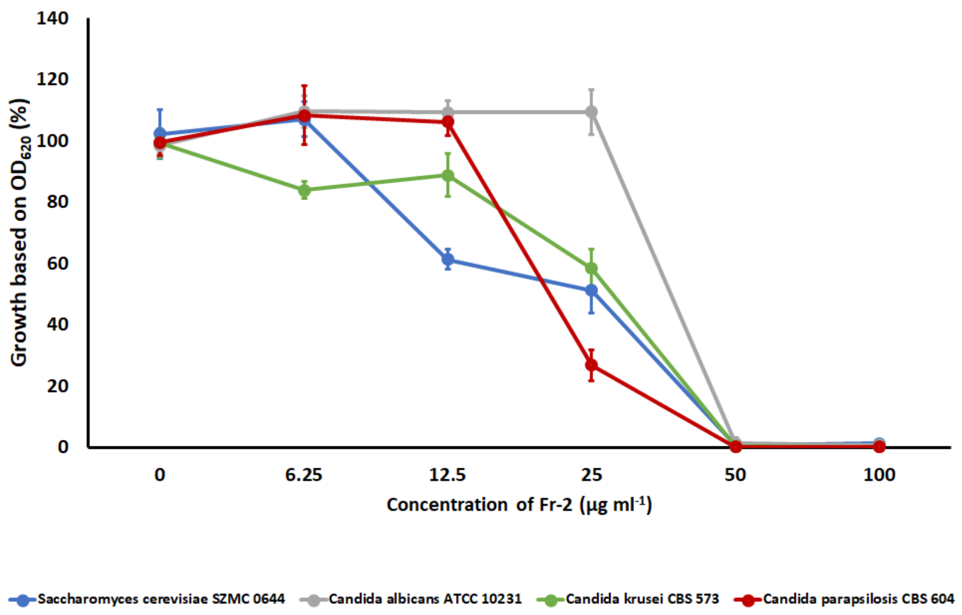

Growth based on OD620 (%)
140
120
100
80
60
40
20
0
0
6.25
12.5
25
50
100
Concentration of Fr-2 (µg ml-1)
Saccharomyces cerevisiae SZMC 0644
Candida albicans ATCC 10231
Candida krusei CBS 573
Candida parapsilosis CBS 604

B

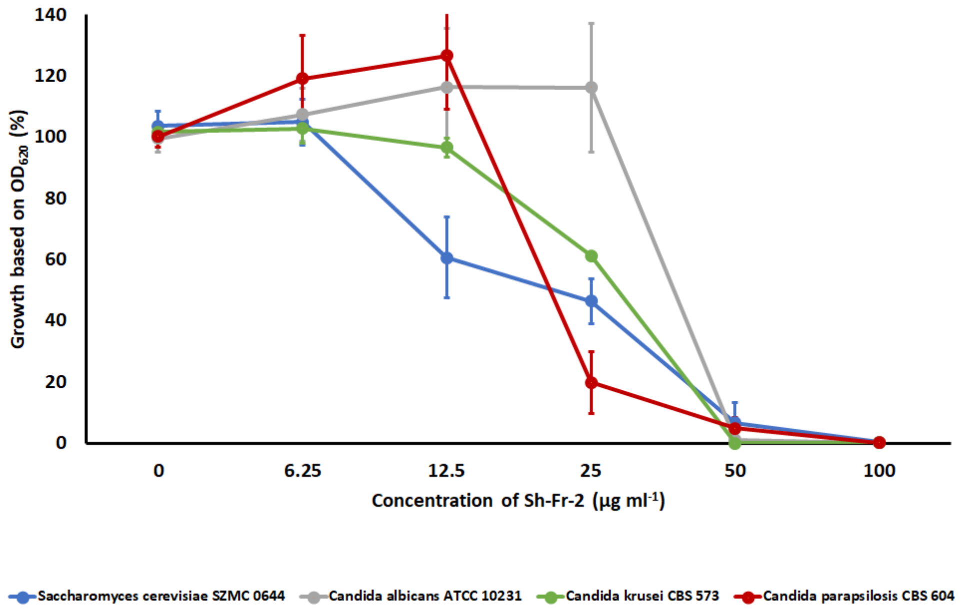

Growth based on OD620 (%)
140
120
100
80
60
40
20
0
0
6.25
12.5
25
50
100
Concentration of Sh-Fr-2 (µg ml-1)
Saccharomyces cerevisiae SZMC 0644
Candida albicans ATCC 10231
Candida krusei CBS 573
Candida parapsilosis CBS 604

C

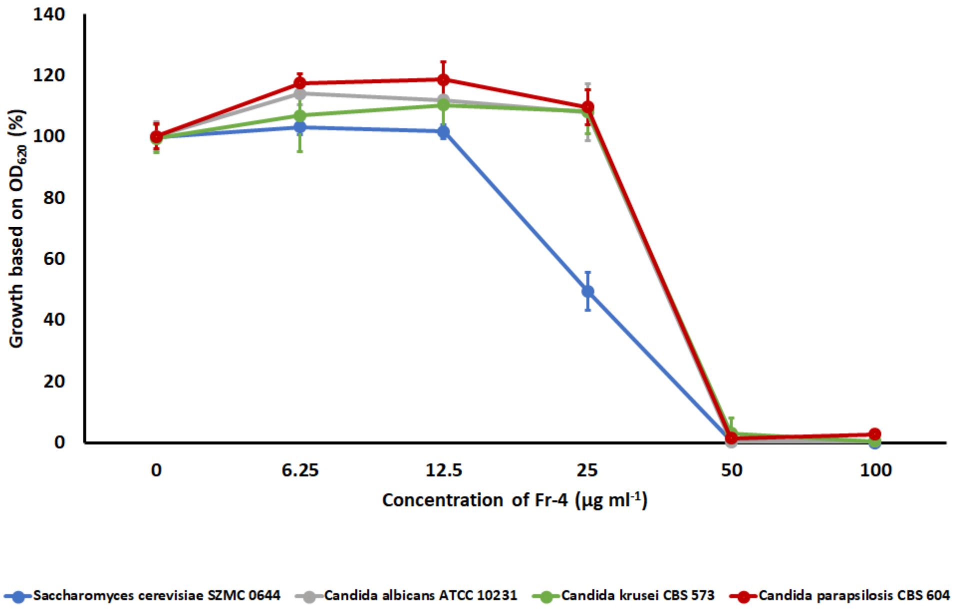

D

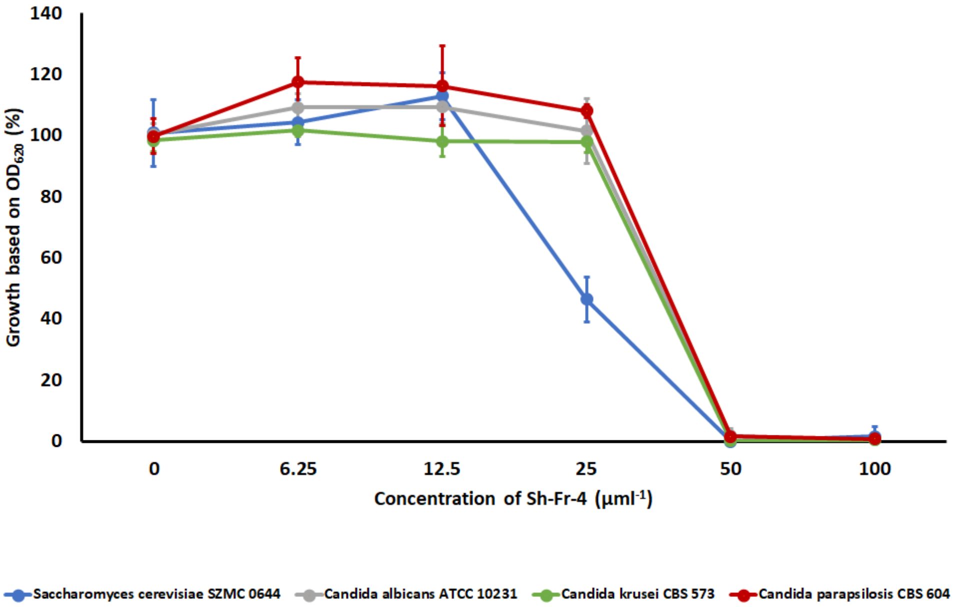

Supplementary Figure 6. Growth inhibition potential of **(A)** Fr-2, **(C)** Fr-4 of NFAP2 and their shuffled variants **(B)** Sh-Fr- 2 and *(D)* Sh-Fr-4 at their different concentrations in LCM after incubation for 48 hours at 30 °C. Fr-2 and 4: synthetic peptide Fragment 2 and 4 of NFAP2 (**Table 1**); Sh-Fr-2 and 4: shuffle variants of synthetic peptide Fragments 2 and 4 of NFAP2 (**Table 1**).

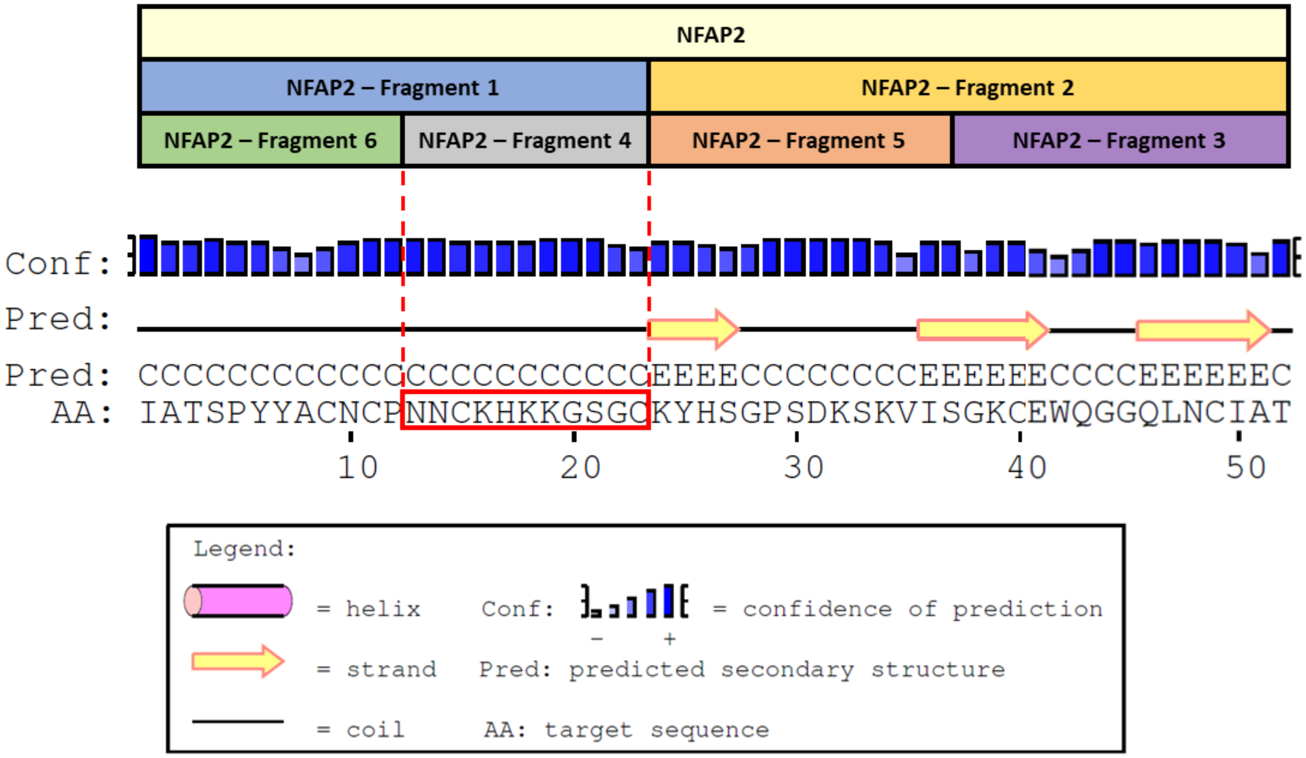

Supplementary Figure 7. Putative secondary structure of NFAP2 by the by PSIPRED (v3.3) Protein Analysis Workbench (Buchan et al., 2013). Fragment 4 (**Table 1**), the predicted functional active part of NFAP2 is indicated in red box.

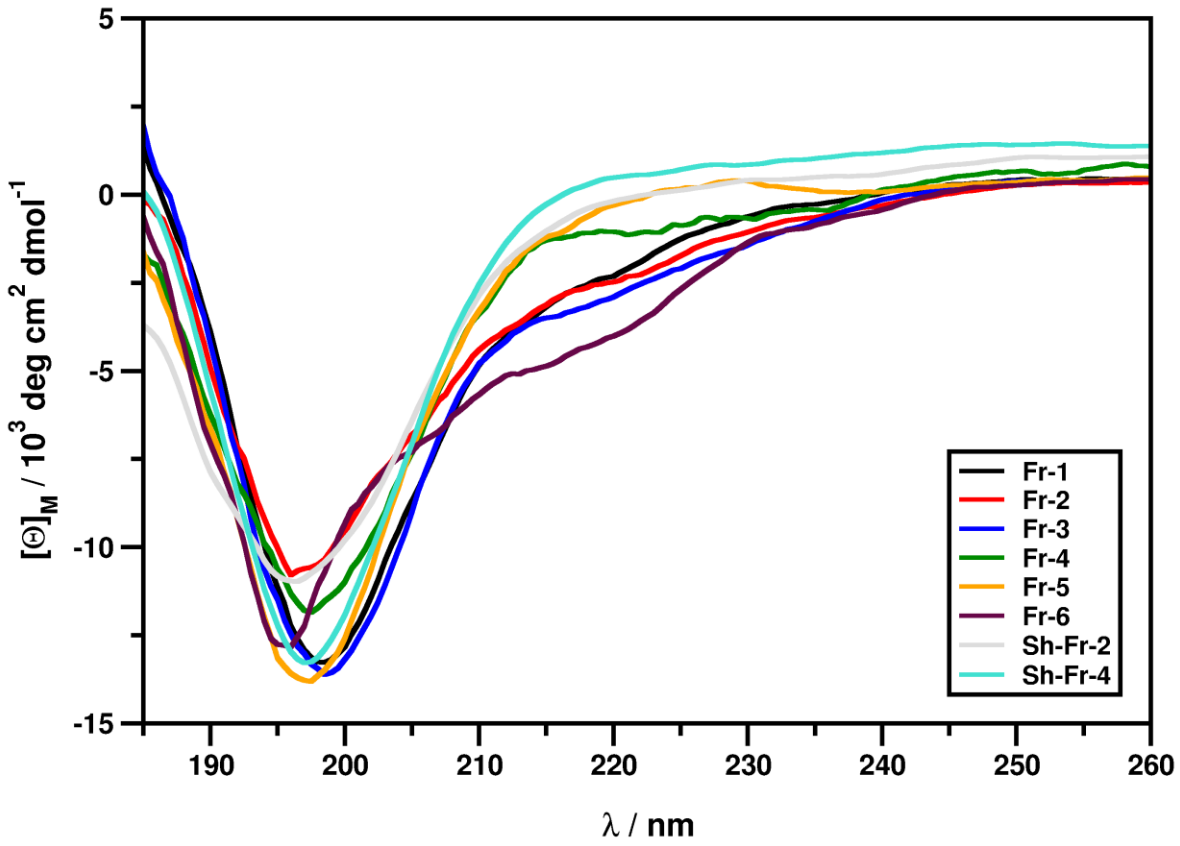

Supplementary Figure 8. ECD spectra of synthetic NFAP2 peptide fragments recorded at 25 °C. Fr-1 - 6: synthetic peptide Fragment 1 - 6 of NFAP2 (**Table 1**), Sh-Fr-2 and 4: shuffle variants of synthetic peptide Fragment 2 and 4 of NFAP2 (**Table 1**).
